# Supplementary material for: Two-layer Electrospun System Enabling Wound Exudate Management and Visual Infection Response
Source: Sensors (Basel). 2019 Feb 26;19(5):991. doi: 10.3390/s19050991 (PMC6427107; doi:10.3390/s19050991)
Supplement: Supplementary file 1 [file sensors-19-00991-s001.pdf]

Supplementary material

An infection-responsive electrospun nanofibrous membrane with integrated colour change capability

Mohamed Basel Bazbouz [a], Giuseppe Tronci [a, b]

[a] Textile Technology Research Group, School of Design, University of Leeds, UK
[b] Biomaterials and Tissue Engineering Research Group, School of Dentistry, St. James's University Hospital, University of Leeds, UK

Correspondence: m.b.bazbouz@leeds.ac.uk (M.B.B.), g.tronci@leeds.ac.uk (G.T.)

Supplementary material

Fig. S1: Photograph of FSES-based scalable manufacture of the electrospun double-layered nanofibrous membranes.

Fig. S2: The SEM morphology of FS electrospun PAA before crosslinking at magnification of 2000x.

Fig. S3: The SEM morphology of FS electrospun PAA before crosslinking at magnification of 10000x.

Fig. S4: The SEM morphology of FS electrospun PAA after crosslinking at magnification of 5000x.

Fig. S5: The SEM morphology of FS electrospun PAA after crosslinking at magnification of 10000x.

Fig. S6: The SEM morphology of FS electrospun PMMA-co-MAA at magnification of 500x.

Fig. S7: The SEM morphology of FS electrospun PMMA-co-MAA at magnification of 10000x.

Fig. S8: The response of BTB when immersed in different pH buffer solutions.

Fig. S9: The response of the hybrid nanofibrous membrane PAA*(PMMA-co-MAA)10 to the increase of pH values at different buffer solutions, immediately after soaking.

Fig. S10: The response of the hybrid nanofibrous membrane PAA*(PMMA-co-MAA)10 to the increase of pH values at different buffer solutions after two-hour incubation.

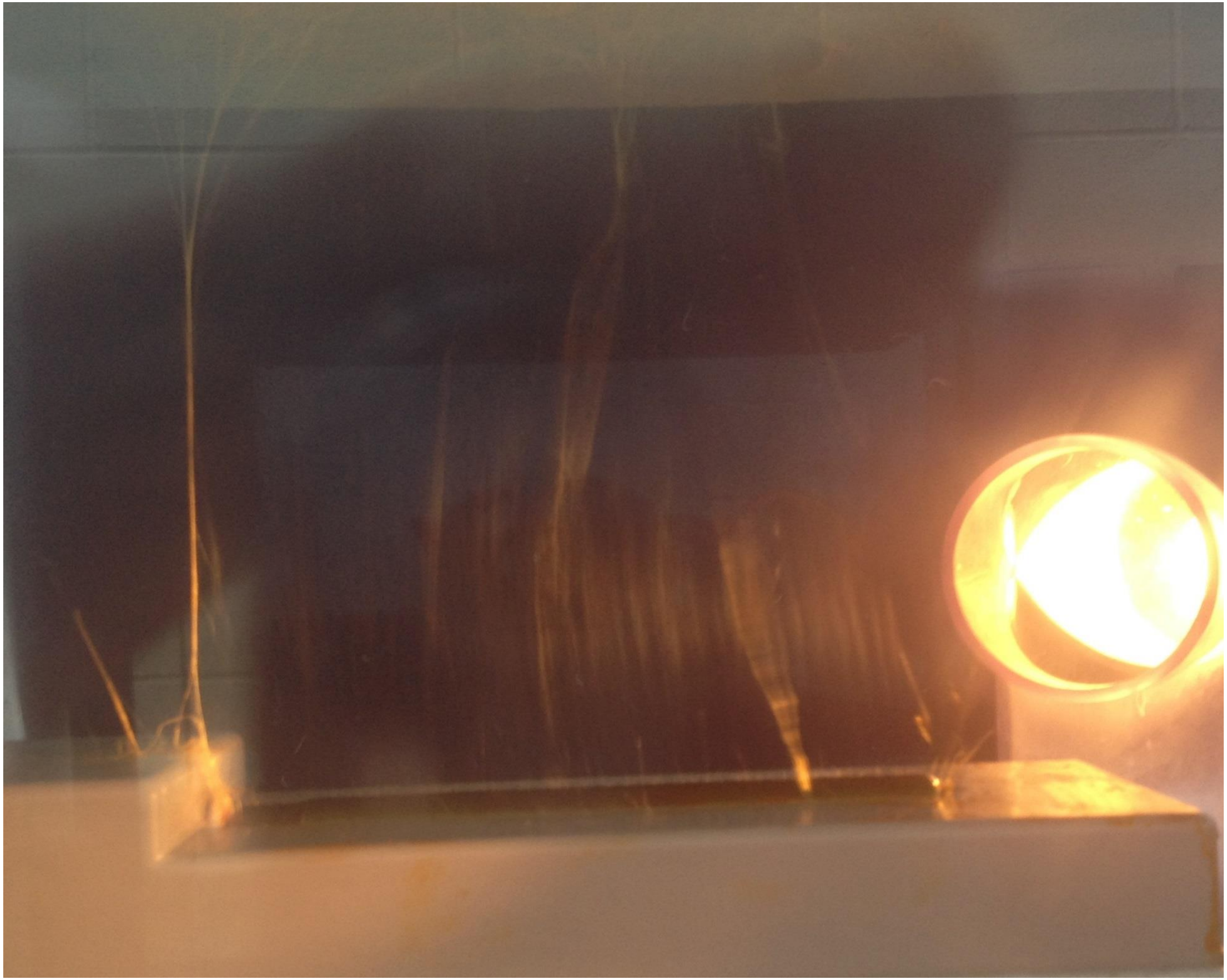

Fig. S1: Photograph of FSES-based scalable manufacture of the electrospun double-layered nanofibrous membranes.

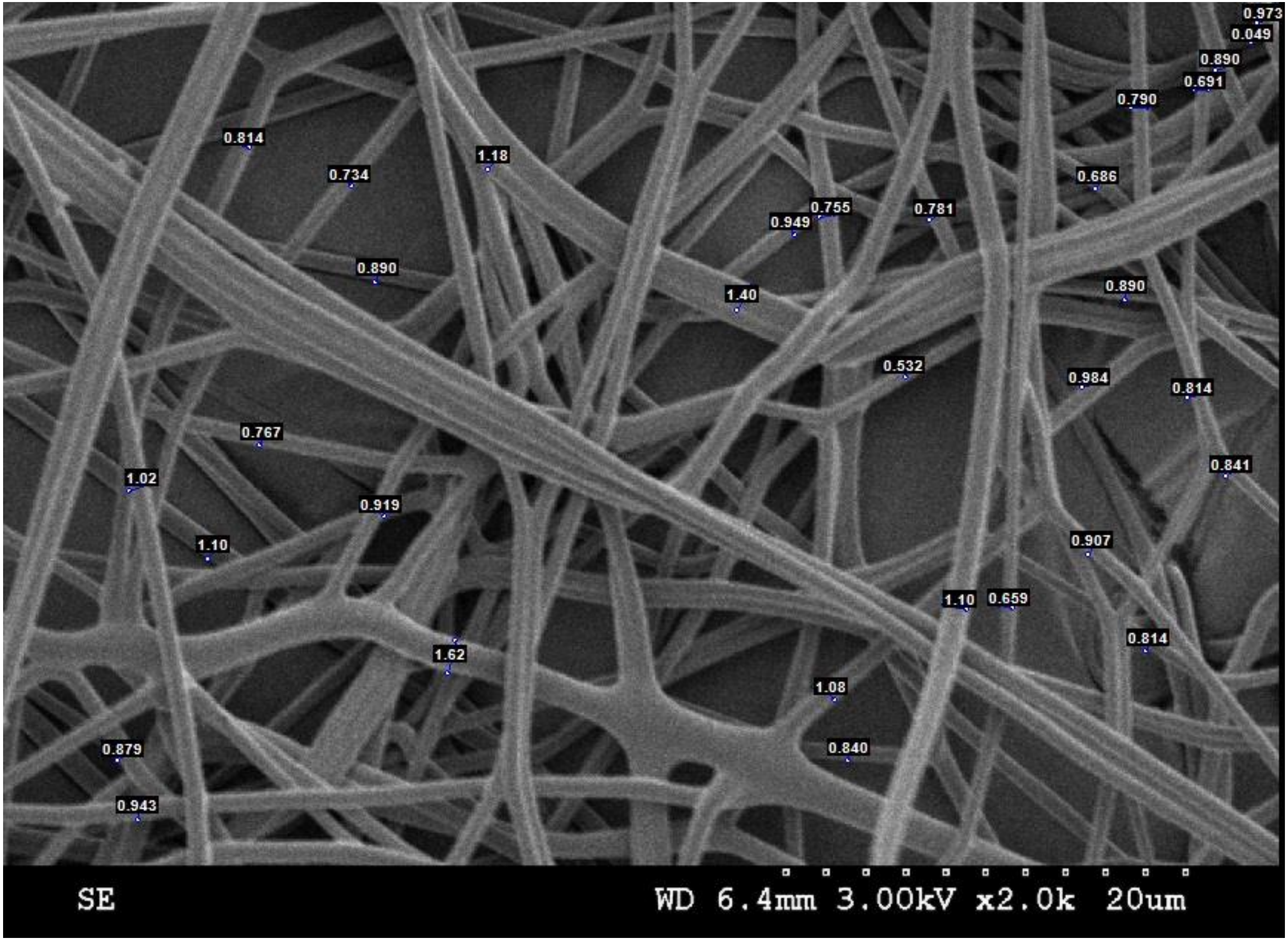

Fig. S2: The SEM morphology of FS electrospun PAA before crosslinking at magnification of 2000x.

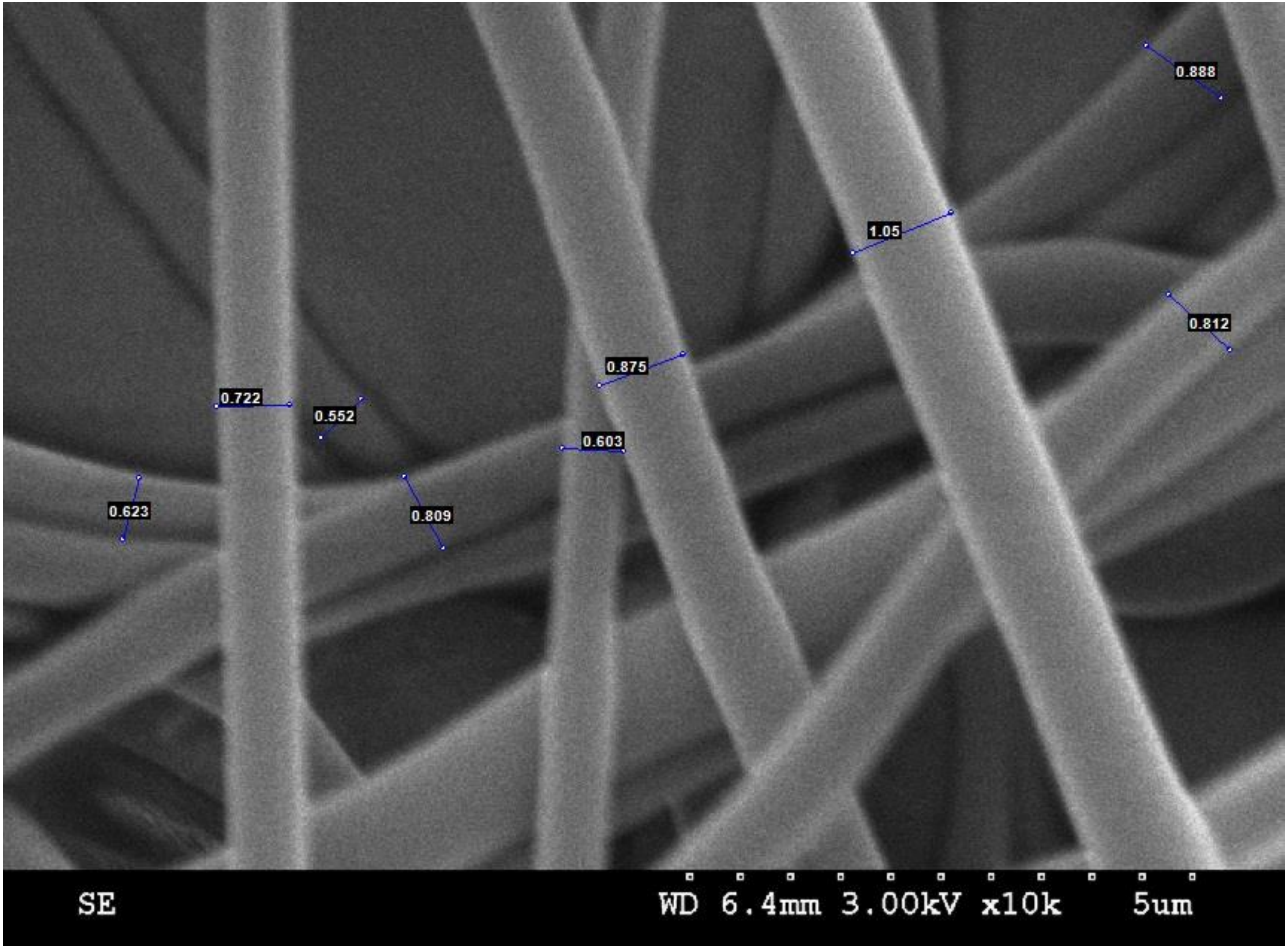

Fig. S3: The SEM morphology of FS electrospun PAA before crosslinking at magnification of 10000x.

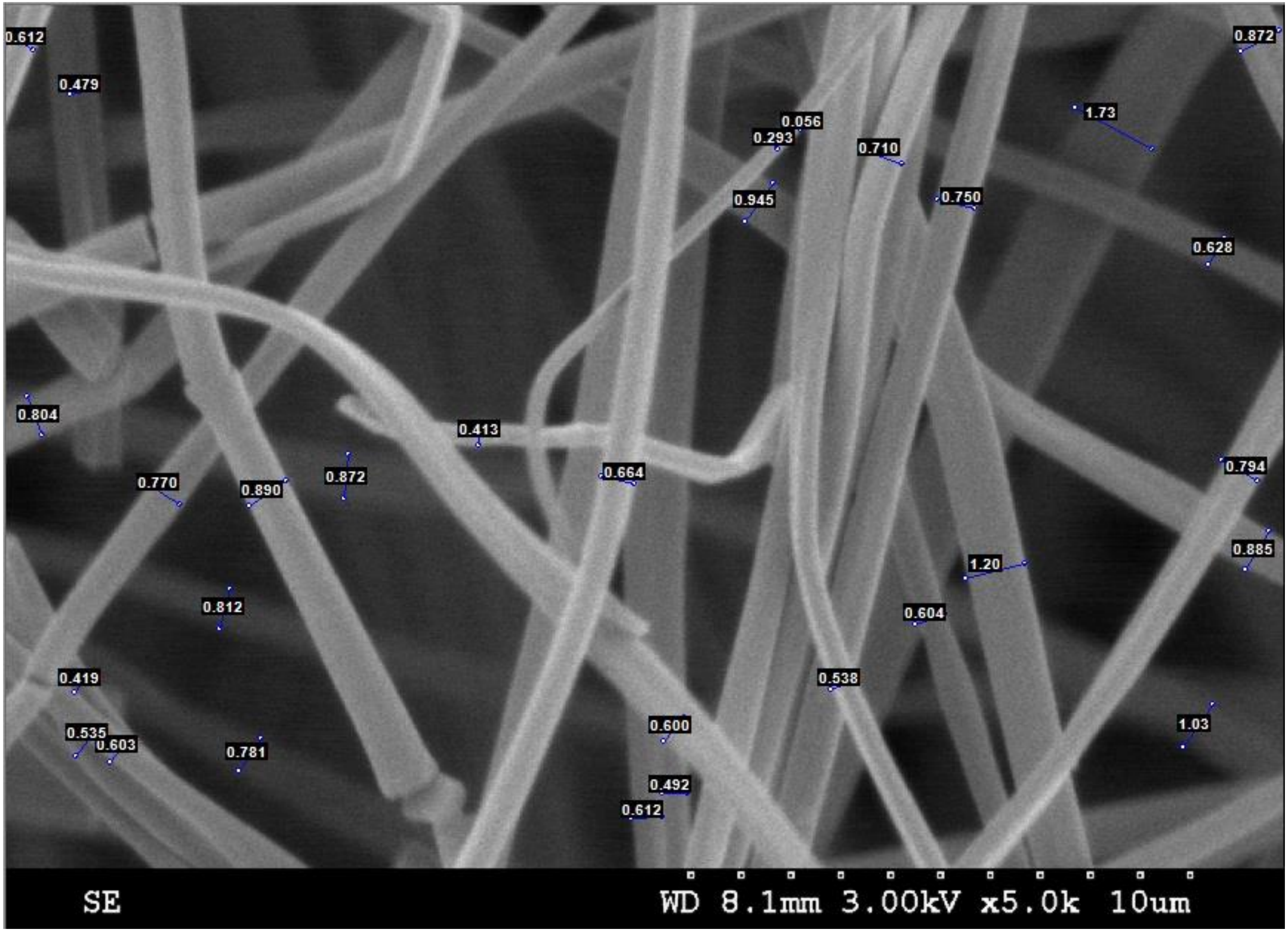

Fig. S4: The SEM morphology of FS electrospun PAA after crosslinking at magnification of 5000x.

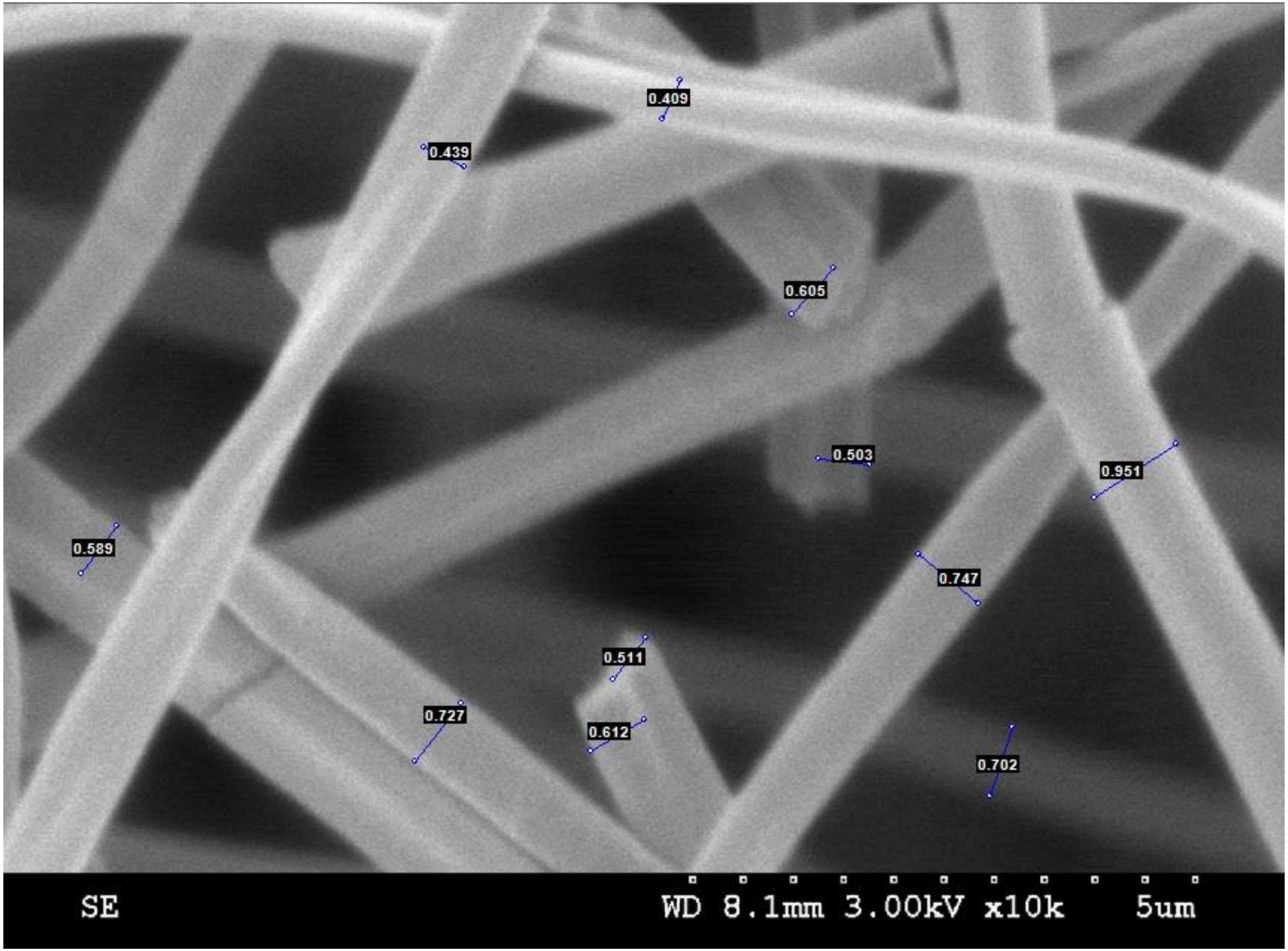

Fig. S5: The SEM morphology of FS electrospun PAA after crosslinking at magnification of 10000x.

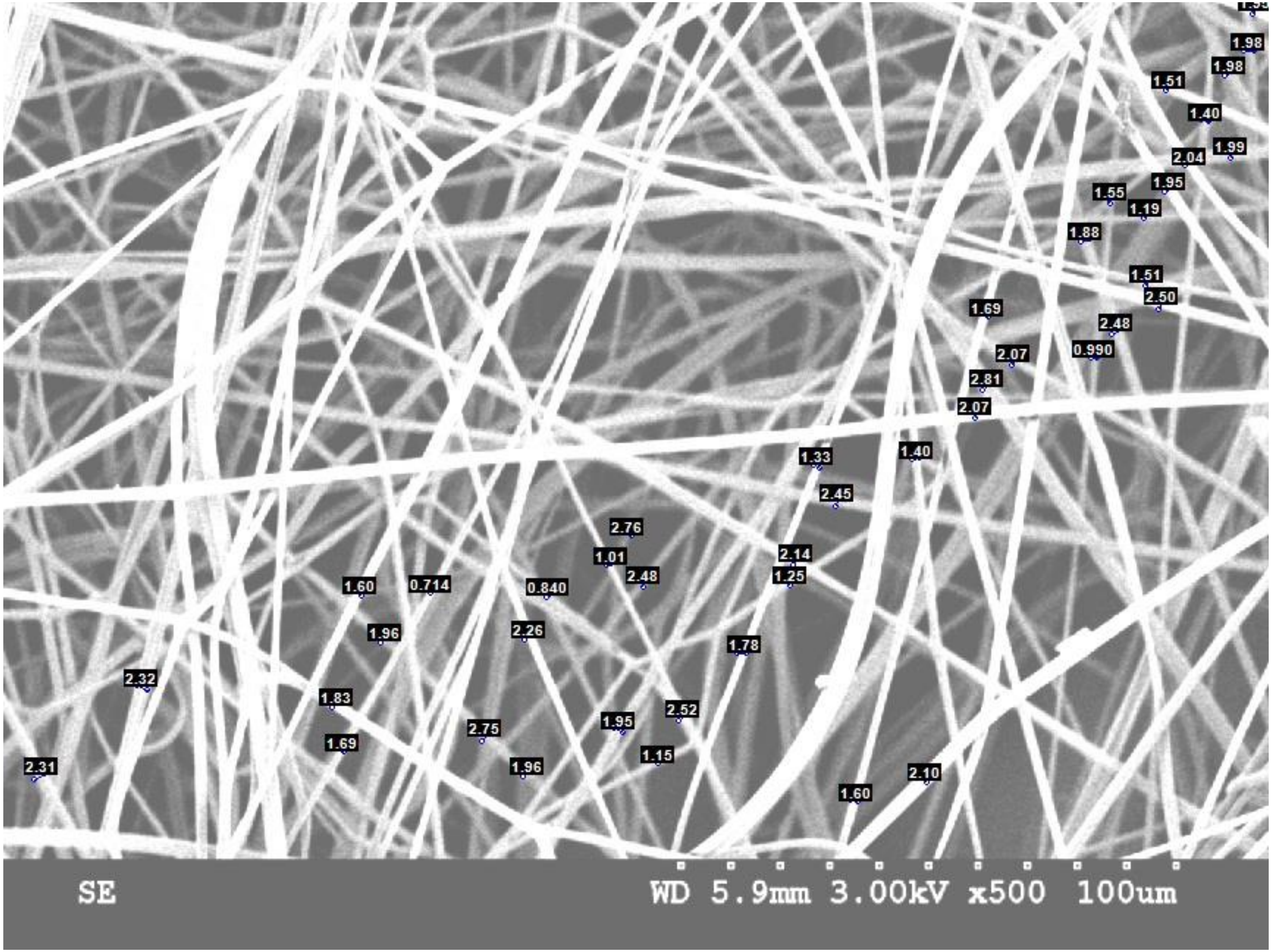

Fig. S6: The SEM morphology of FS electrospun PMMA-co-MAA at magnification of 500x.

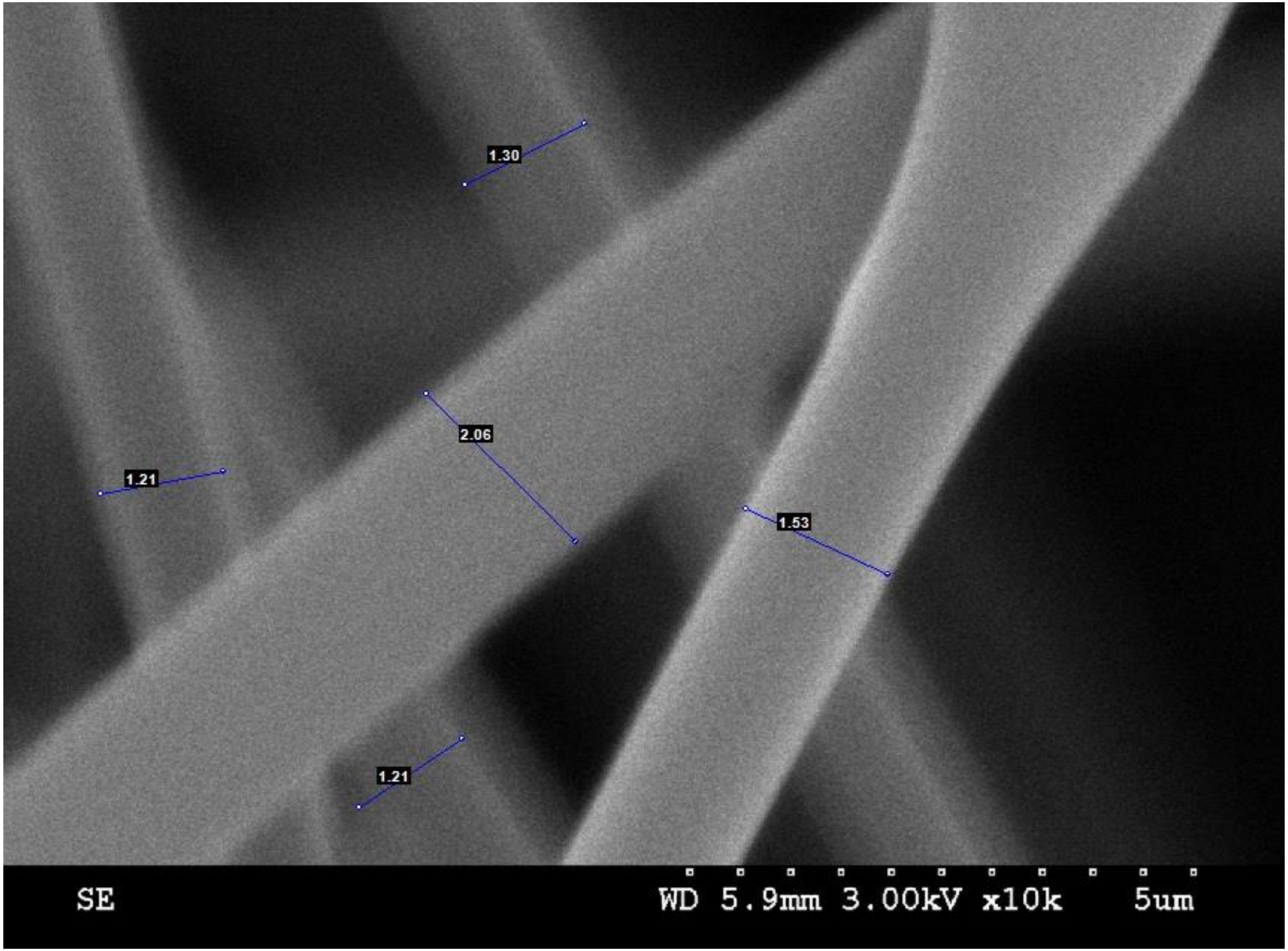

Fig. S7: The SEM morphology of FS electrospun PMMA-co-MAA at magnification of 10000x.

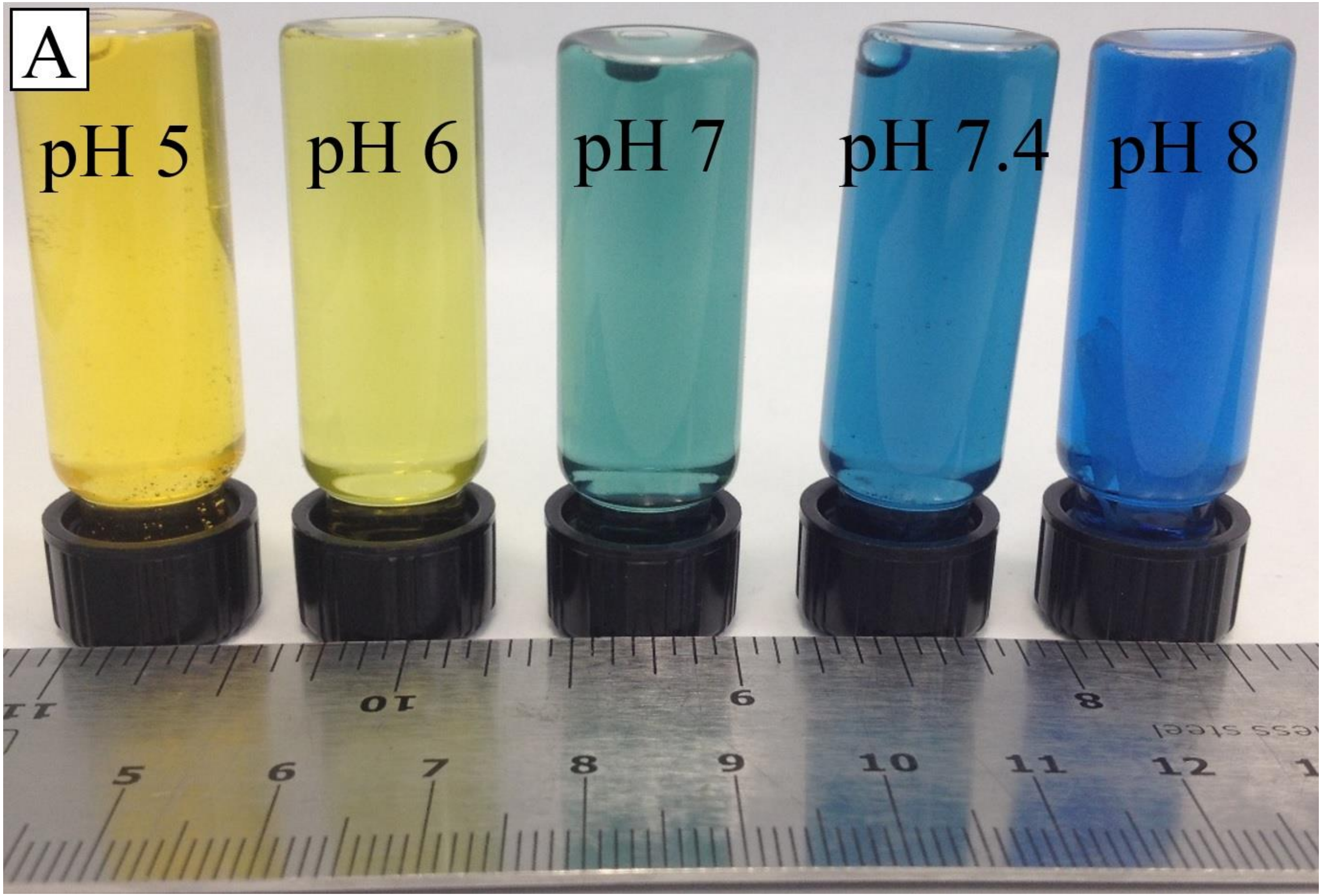

Fig. S8: The response of BTB when immersed in different pH buffer solutions.

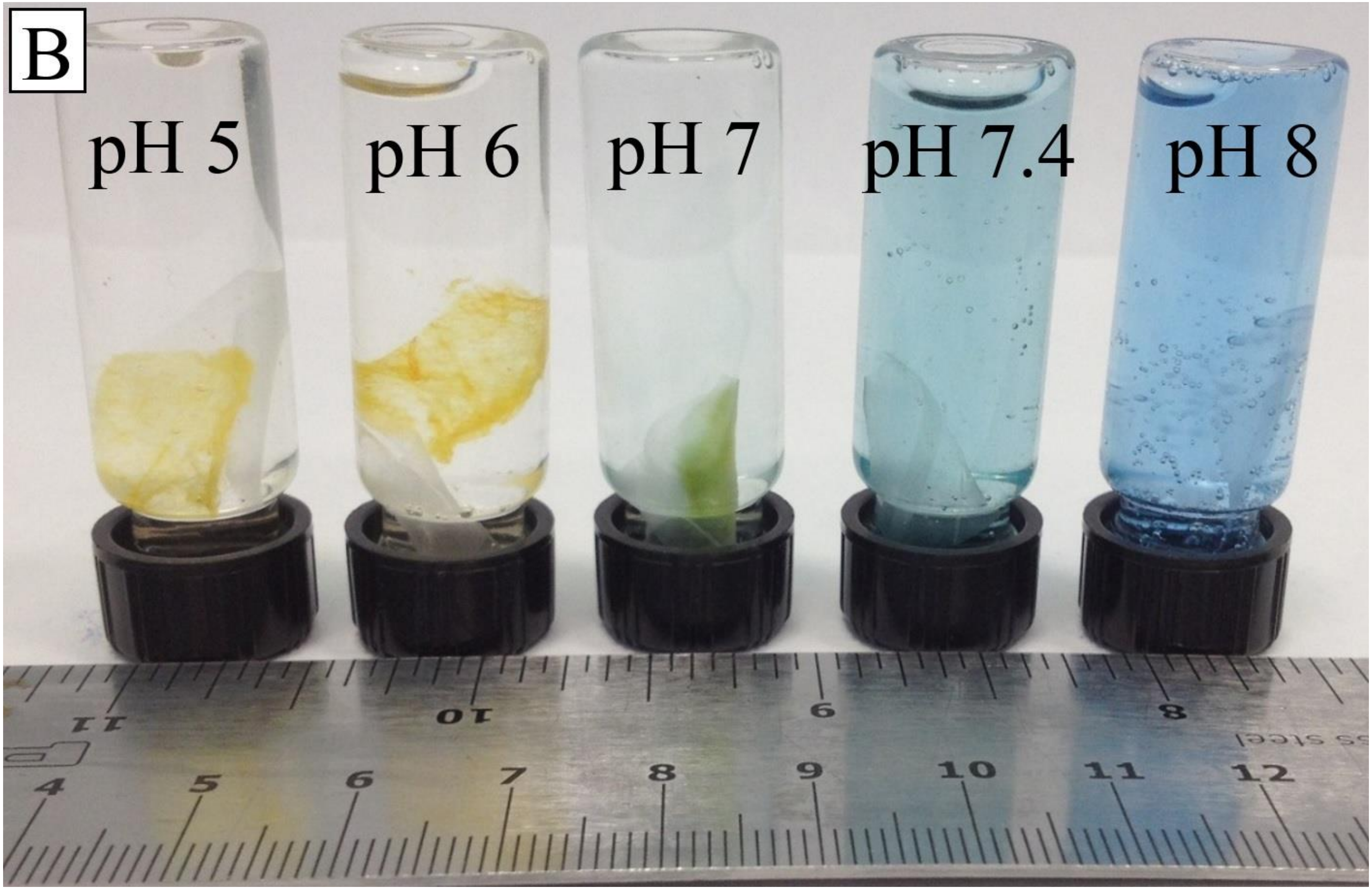

Fig. S9: The response of the hybrid nanofibrous membrane PAA*(PMMA-co-MAA)10 to the increase of pH values at different buffer solutions, immediately following contact with incubation.

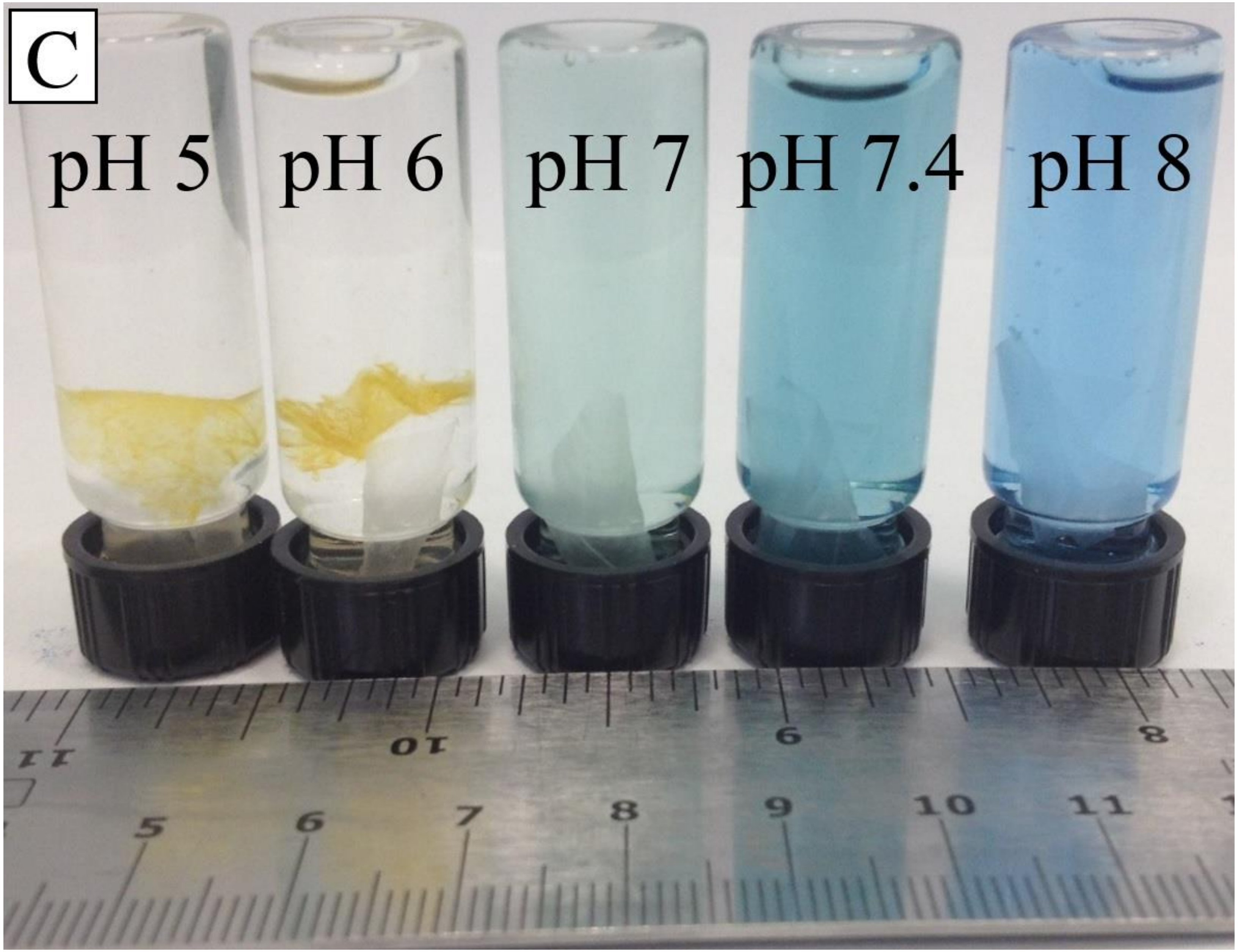

Fig. S10: The response of the hybrid nanofibrous membrane PAA*(PMMA-co-MAA)10 to the increase of pH values at different buffer solutions after two-hour incubation.
